# Giant Pleomorphic Adenoma of the Parotid Gland: Surgical Management of a Long‐Standing Tumor With Facial Deformity and Preservation of Facial Nerve Function

**DOI:** 10.1155/crot/7531403

**Published:** 2026-10-15

**Authors:** Francesco Ferragina, Angelo R. Sottile, Giuseppe Tarallo, Maria Grazia Ioppolo, Maria Giulia Cristofaro

**Affiliations:** ^1^ Department of Experimental and Clinical Medicine Maxillofacial Surgery Unit Renato Dulbecco Hospital Magna Graecia University of Catanzaro Viale Europa Catanzaro 88100 Italy unicz.it; ^2^ Department of Neurosciences Maxillofacial Surgery Operative Unit Reproductive and Odontostomatological Sciences Federico II University of Naples Naples 80131 Italy unina.it

**Keywords:** benign salivary gland tumor, facial asymmetry, giant parotid tumor, head and neck surgery, surgical excision

## Abstract

**Background:**

Pleomorphic adenoma (PA), the most prevalent benign neoplasm of the parotid gland, typically manifests as a slow‐growing, painless mass. In cases where treatment is substantially delayed, the tumor may undergo progressive enlargement, potentially resulting in significant facial deformity. This, in turn, can complicate surgical management.

**Case Presentation:**

A 60‐year‐old female patient exhibited a long‐standing right parotid mass that had been gradually increasing in size since 2007, with a notable escalation in dimensions during the year prior to the preliminary assessment. Magnetic resonance imaging in 2021 revealed a well‐defined, multilobulated right parotid lesion measuring over 9 cm, and fine‐needle aspiration cytology was consistent with PA. Despite the recommendation of surgical excision, the patient initially declined treatment and was subsequently lost to follow‐up. In 2025, the patient returned with a parotid mass measuring over 15 cm, accompanied by pronounced facial asymmetry and significant skin stretching. The patient underwent a superficial parotidectomy with complete excision of the lesion, meticulous monitoring of the facial nerve, and preservation of its function. Concurrently, surgical interventions were performed to modify the patient’s skin. A subsequent histopathological examination confirmed the presence of PA, devoid of any substantial nuclear atypia or augmented mitotic activity. On Postoperative Day 3, a hematoma developed at the surgical site, necessitating surgical evacuation and hemostatic revision. The ensuing recovery was uneventful, and follow‐up at 14 months postoperatively confirmed no recurrence and complete facial nerve function.

**Conclusions:**

This case exemplifies the ramifications of protracted untreated growth of a PA, culminating in substantial tumor enlargement and pronounced facial deformity. Notwithstanding the advanced size of the lesion, complete excision was achieved while preserving the function of the facial nerve. In the management of giant parotid tumors, meticulous surgical planning and close postoperative monitoring are paramount.

## 1. Introduction

Pleomorphic adenoma (PA) is the most prevalent benign tumor of the salivary gland, with the parotid gland being the site of origin in many cases [1–4]. Typically, it manifests as a slow‐growing, painless, and well‐circumscribed mass. While most PAs are diagnosed and treated before reaching a substantial size, it is noteworthy that untreated lesions have the potential to undergo progressive enlargement. In rare instances, these lesions may evolve into giant tumors that exceed 10 cm in their greatest dimension [1–6].

Giant parotid PAs pose a unique surgical challenge due to their capacity for progressive enlargement, which can lead to significant facial asymmetry, substantial stretching and remodeling of the overlying skin, and distortion of the anatomical relationships within the parotid region. These changes have the potential to introduce complexity in the processes of tumor exposure, dissection, and facial nerve identification and preservation. Consequently, surgical intervention necessitates a meticulous preoperative evaluation and a meticulous intraoperative technique, particularly when the tumor has attained an extraordinary size.

The present case is noteworthy for the exceptionally prolonged untreated course of a parotid PA, with documented progression from a 9.8 × 5.2 × 4 cm lesion in 2021 to a giant mass exceeding 15 cm in 2025 after the patient initially declined surgical treatment and was subsequently lost to follow‐up. The tumor ultimately resulted in significant facial deformity and substantial soft‐tissue distortion. Notwithstanding the anatomical changes and the remarkable dimensions of the tumor, complete surgical excision was accomplished, accompanied by the preservation of facial nerve function. Concomitant surgical remodeling of the overlying skin was also necessary. The postoperative course was complicated by the development of a hematoma, which required surgical evacuation and hemostatic revision. A subsequent histopathological examination confirmed a benign PA, devoid of any significant nuclear atypia or increased mitotic activity. It is understood that the combination of prolonged untreated growth, documented enlargement to giant dimensions, marked facial deformity, successful facial nerve preservation, skin remodeling, and management of a postoperative hematoma renders this case a clinically relevant example of the challenges associated with delayed treatment of giant parotid PA.

## 2. Case Presentation

A 60‐year‐old female patient was referred to the Maxillofacial Surgery Unit of the University of Catanzaro in October 2021 due to a right preauricular/parotid swelling that had been in existence since 2007. According to the patient’s medical history, the mass gradually increased in size over approximately 14 years. In the year before the specialist’s preliminary evaluation, the swelling grew markedly faster. Her medical history was notable for hypertension and anxiety‐depressive syndrome.

### 2.1. Preliminary Clinical Evaluation and Diagnostic Assessment

A painless mass measuring approximately 10 × 6 cm was observed in the right parotid/preauricular region during the clinical examination in 2021. The lesion exhibited a smooth surface, regular margins with tensile elasticity, and minimal mobility in relation to the superficial and deep planes (Figure 1). The overlying skin was found to be intact, and no clinically evident facial nerve dysfunction was observed (Grade I according to the House–Brackmann scale). A cervical lymph node examination and an oral examination were conducted, both of which yielded negative results for the presence of ongoing focal lesions. The patient’s mouth opening, swallowing, and airway function were all found to be within normal limits. No hearing complaints or other cranial nerve deficits were noted. An intraoral examination revealed no bulging of the lateral pharyngeal wall.

**FIGURE 1:**
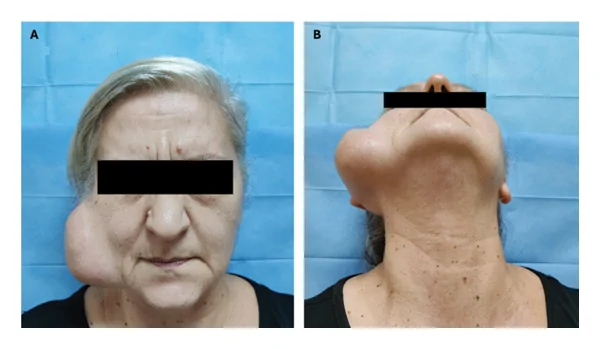
The (A) frontal and (B) submental vertex views showing the right parotid‐region swelling.

Magnetic resonance imaging (MRI) performed in December 2021 revealed a well‐defined, multilobulated mass measuring 9.8 × 5.2 × 4.0 cm, arising from the superficial lobe of the right parotid gland and adjacent to the masseter muscle. The facial nerve plane could not be directly visualized but was not displaced beyond the expected anatomical course. There was no radiological evidence of parapharyngeal space extension, no pathologically enlarged cervical lymph nodes, and no evidence of skin or subcutaneous infiltration. The lesion exhibited heterogeneous contrast enhancement and areas of restricted diffusion on diffusion‐weighted imaging (Figure 2).

**FIGURE 2:**
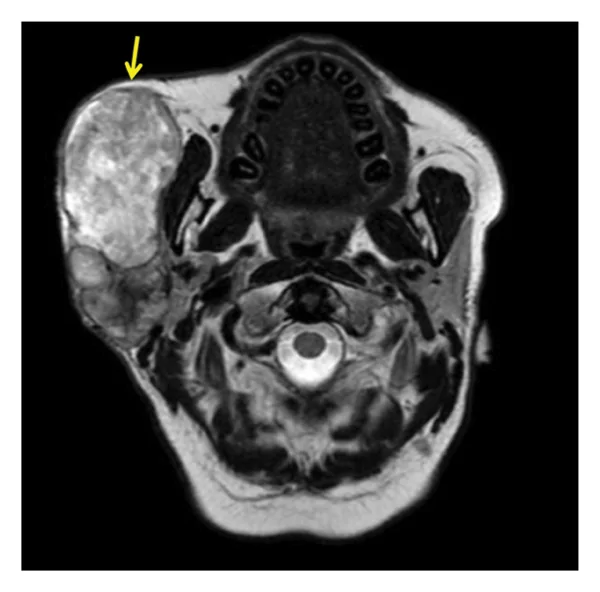
Axial contrast–enhanced MRI demonstrating a well‐defined, multilobulated mass involving the upper portion of the right parotid gland. The lesion is indicated by the arrow.

Fine‐needle aspiration cytology (FNAC) performed in December 2021 revealed a diagnosis consistent with PA. The cytological findings included epithelial and myoepithelial cells arranged in cohesive clusters and singly dispersed within abundant fibrillary chondromyxoid stroma. The nuclei were round to oval and exhibited no significant cytological atypia. The provisional diagnosis was a giant PA, subsequently supported by FNAC.

Given the progressive enlargement of the lesion, surgical excision was recommended. However, the patient refused the recommended surgical intervention and subsequently failed to attend the scheduled clinical follow‐up visits.

### 2.2. Clinical and Radiological Progression

The patient returned to our institution in March 2025, approximately 3 years after the initial diagnostic evaluation. Concurrently, a conspicuous augmentation in the dimensions of the tumor was manifestly evident on clinical examination. The mass exhibited a dimension greater than 15 cm in its most prominent clinical dimension and manifested with pronounced facial asymmetry. The overlying skin exhibited signs of tension and telangiectasia, while the lesion demonstrated increased firmness and an apparent adherence to the surrounding superficial and deep planes. The patient continued to demonstrate absence of pain and no clinically evident facial nerve dysfunction (Grade I according to the House–Brackmann scale) (Figure 3).

**FIGURE 3:**
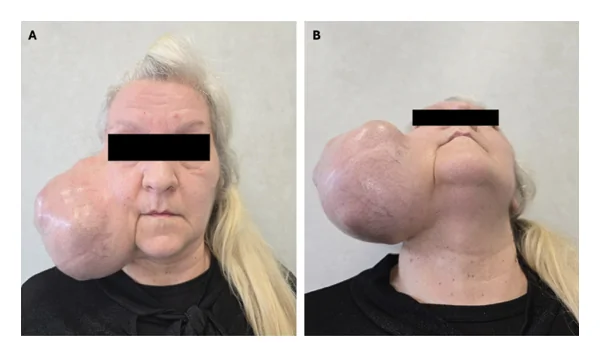
The (A) frontal and (B) submental vertex views almost 4 years from the time of her surgery refusal.

A contrast‐enhanced computed tomography (CT) scan of the head and neck revealed a substantial right parotid‐region mass measuring approximately 14.5 cm in its greatest dimension. The lesion exhibited a relatively well‐defined margin but demonstrated heterogeneous density and predominantly peripheral contrast enhancement, accompanied by extensive internal necrotic and inflammatory‐appearing areas. No significant involvement of the adjacent bone, muscle, vascular structures, or major neural structures was reported (Figure 4). The lesion appeared to arise predominantly from the superficial lobe, without evidence of parapharyngeal space extension or transcervical/deep lobe dumbbell configuration. No pathologically enlarged regional lymph nodes were identified. The overlying skin showed stretching but no radiological signs of dermal infiltration.

**FIGURE 4:**
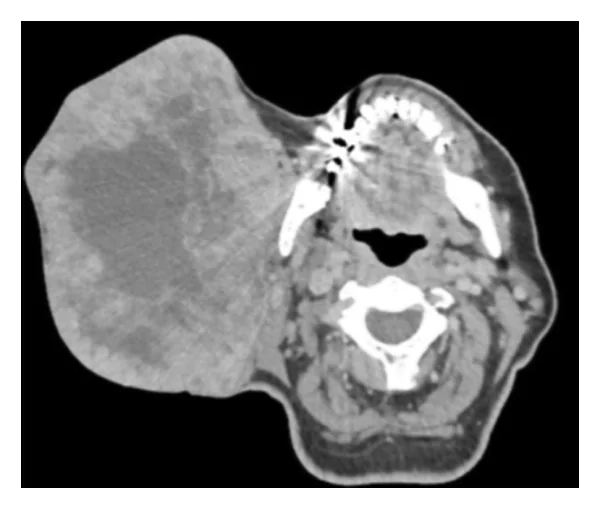
Axial contrast–enhanced CT demonstrating the giant right parotid region mass with heterogeneous internal density and peripheral enhancement.

The discrepancy between the measurements obtained at the various stages should be interpreted as documented tumor progression rather than as directly interchangeable measurements. Specifically, the lesion exhibited dimensions of 9.8 × 5.2 × 4.0 cm on MRI in December 2021, approximately 14.5 cm on CT in March 2025, and exceeded 15 cm on clinical examination at the same time point. Subsequent measurement of the resected specimen yielded dimensions of 16 × 13.5 × 12 cm. It is important to note that these measurements were obtained using different methods and describe different stages of tumor assessment. Therefore, they should not be considered directly equivalent.

### 2.3. Surgical Treatment

In March 2025, the patient underwent surgical excision of the giant right parotid tumor under general anesthesia (Figure 5A). A modified Blair incision was used, and a standard cervicofacial flap was elevated in the subplatysmal/subfascial plane. Given the lesion’s benign nature and extrinsic growth pattern, a superficial (extracapsular) parotidectomy with conservative facial nerve dissection was performed. The main trunk of the facial nerve was identified first, using the tragal pointer and the posterior belly of the digastric muscle as landmarks, and was preserved along its full peripheral course despite close apposition to the tumor capsule; no direct nerve invasion was noted. An intraoperative monitoring system was used to check the integrity of the facial nerve during surgery. The tumor capsule remained macroscopically intact throughout dissection, with no evidence of intraoperative tumor spillage. As the lesion was confined to the superficial lobe, no deep lobe dissection was required. No intraoperative complications occurred. Two closed‐suction drains were placed before wound closure.

**FIGURE 5:**
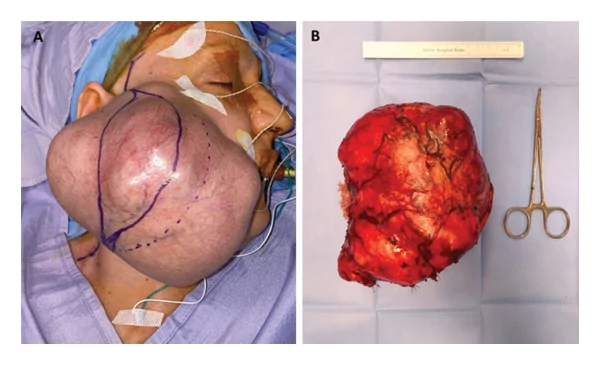
Intraoperative macroscopic views. (A) Image of the patient before surgery. (B) The specimen collected was darker in color than the surrounding gland, and its cut surface demonstrated a firm consistency.

Due to the substantial stretching and remodeling of the overlying skin, excision of redundant or pathologically stretched skin and surgical skin remodeling were necessary. The specimen measured 16 × 13.5 × 12 cm. Gross examination of the cut surface revealed areas of necrosis and hemorrhage, as depicted in Figure 5B.

Immediately postoperatively, facial nerve function was House–Brackmann Grade I.

### 2.4. Postoperative Course

The initial postoperative course was uneventful during the first 48 h, with only mild local edema and no clinical evidence of infection or fluid collection. On Postoperative Day 3, the patient exhibited hypertensive peaks that proved refractory to the initial antihypertensive regimen, accompanied by a new fluctuant swelling at the surgical site, clinically consistent with a postoperative hematoma.

Consequently, the patient was readmitted to the operating room for the removal of the hematoma and the surgical revision of the operative field. Intraoperatively, bleeding points were identified and cauterized throughout the surgical bed, and additional hemostatic material was applied. Subsequent modifications were made to the antihypertensive treatment, and the patient’s clinical course was subsequently deemed uneventful.

### 2.5. Histopathological Examination and Follow‐Up

A subsequent histopathological examination confirmed a PA. Gross examination showed an encapsulated specimen measuring 16 × 13.5 × 12 cm and weighing 1.76 kg, with a grossly intact capsule and margins free of tumor on macroscopic inspection. The lesion exhibited biphasic/triphasic proliferation of epithelial and myoepithelial cells within a variably myxoid and fibrous stromal background. No significant nuclear atypia or increased mitotic activity was identified. The tumor showed moderate cellularity with focal areas of necrosis and hemorrhage, without capsular penetration or satellite nodules. The surgical intervention involved the complete removal of the tumor, and the surgical margins were found to be free of satellite nodules, perineural invasion, and vascular invasion. The areas described as necrotic and inflammatory on radiological imaging did not show signs of malignant transformation, as confirmed by histopathological analysis; the findings did not demonstrate carcinoma ex PA or another malignant salivary gland neoplasm [7]. Features arguing against malignant transformation included the absence of capsular invasion, stromal/perineural/lymphovascular invasion, cytologic atypia, and abnormal mitotic activity. Immunohistochemistry for p53 and Ki‐67 was performed to further exclude malignant transformation, showing low and homogeneous indices. To rule out the possibility of a salivary ductal carcinoma component, HER2 was evaluated and tested negative.

Approximately 3 months after the surgical procedure, a clinical examination was conducted, which revealed that the healing process was proceeding satisfactorily and that the function of the facial nerve had been maintained (Grade I according to the House–Brackmann scale) (Figures 6A and B). A subsequent ultrasound performed at 3 months revealed no evidence of local recurrence or pathological cervical lymphadenopathy. The patient is to remain under both clinical and instrumental surveillance.

**FIGURE 6:**
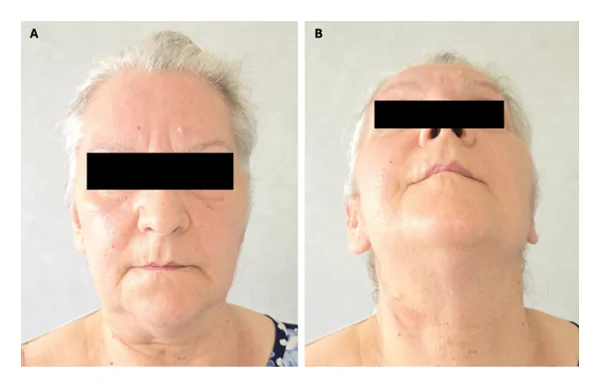
The (A) frontal and (B) submental vertex views after almost 3 months of surgery.

A further clinical follow‐up visit was performed in May 2026, approximately 14 months after surgery, confirming an excellent postoperative course with no clinical evidence of tumor recurrence and complete preservation of facial nerve function (House–Brackmann Grade I). The patient is to remain under both clinical and instrumental surveillance.

## 3. Discussion

Giant PAs of the parotid gland (> 10 cm) are exceptionally rare, as most PAs are diagnosed and treated at an early stage [1–4]. Delayed intervention, often driven by patient apprehension or loss to follow‐up, can result in massive neoplastic expansion, severe facial deformity, and distortion of regional anatomy, posing distinctive challenges for facial nerve preservation, soft‐tissue reconstruction, and complication management [5, 8–13]. This case documents the natural progression of an untreated parotid PA over 18 years and describes the surgical strategies used to preserve nerve function and manage tissue remodeling.

A key aspect of the present case is the serial documentation of tumor growth over years of delayed surgery. The initial lesion, measured at 9.8 × 5.2 × 4.0 cm on MRI in 2021, exhibited an approximate growth of 14.5 cm on CT and over 15 cm on clinical evaluation in 2025. This progression ultimately resulted in a resected specimen measuring 16 × 13.5 × 12 cm and weighing 1.76 kg. Even though measurements obtained via different methodologies (in vivo cross‐sectional imaging vs. gross physical pathology) reflect distinct physical boundaries and tissue elasticity, the consistent increase across all parameters documents genuine, substantial neoplastic progression during the period of interrupted surveillance. The marked increase in size was associated with considerable stretching and remodeling of the overlying facial skin and consequent facial asymmetry, features that have also been described in previously reported giant PAs [4–6, 10–13].

The long‐standing duration and rapid clinical growth of the parotid mass raised clinical and radiological suspicion of malignant transformation into carcinoma ex PA. However, this remained a suspicion based on imaging alone: the 2025 CT scan showed significant internal heterogeneity, peripheral enhancement, and extensive necrotic/cystic‐appearing areas, features that can also be seen in high‐grade malignancy. Histopathological examination confirmed that these radiological changes corresponded to ischemic necrosis, cystic degeneration, and intratumoral hemorrhage within an entirely benign PA, with no evidence of malignant transformation. The risk of malignant transformation has been reported to increase from approximately 1.6% in tumors present for less than 5 years to 9.5% in those present for more than 15 years [14], with an overall reported incidence ranging from 1.9% to 23.3% [15]. This distinction is clinically important: prolonged tumor duration and rapid growth increase the index of suspicion and justify prompt surgical exploration, but do not by themselves establish an individual patient’s risk of malignancy, which can only be determined by histopathological sampling.

Histopathology is of key importance in the definitive diagnosis of PA due to its striking morphological diversity. As PA exhibits a varied histopathologic presentation, it may be confused histopathologically with myoepithelioma, adenoid cystic carcinoma, mucoepidermoid carcinoma, and basal cell adenoma [9, 16]. Myoepithelioma is characterized by its unique myoepithelial composition and the absence of prominent ductal‐epithelial structures and classic chondromyxoid stroma. Adenoid cystic carcinoma is characterized by distinct morphologic patterns, including cribriform, tubular, or solid, with infiltrative margins and perineural invasion. Notably, it lacks the distinctive myxoid/chondroid background observed in PA. Mucoepidermoid carcinoma is a distinct neoplasm characterized by a unique combination of mucin‐secreting, epidermoid, and intermediate cells, which is distinct from the characteristic stroma observed in papillary adenocarcinoma. Basal cell adenoma is characterized by a monomorphic basaloid pattern, featuring prominent basement membrane–like material and a lack of mesenchyme‐like stromal components. In this instance, the presence of a classic biphasic epithelial–myoepithelial proliferation embedded within a myxoid and fibrous background, in the absence of nuclear atypia, capsular invasion, or augmented mitotic activity, served to confirm the diagnosis of benign PA [8, 16].

Surgical management of giant parotid PAs necessitates meticulous anatomical dissection. The substantial tumor growth exerts significant strain on the facial skin, resulting in the displacement of the primary trunk and peripheral branches of the facial nerve. Notwithstanding the severe anatomical distortion, successful identification and total preservation of the facial nerve were achieved using continuous intraoperative nerve monitoring and anatomical landmarks (the tragal pointer and posterior belly of the digastric muscle). This outcome underscores the notion that the size of the tumor, in and of itself, does not provide sufficient rationale for the sacrifice of the facial nerve, unless there is verification of intraoperative nerve invasion or malignant infiltration. Furthermore, the chronic mechanical stretching of the overlying integument necessitated an elliptical excision of redundant skin, followed by cervicofacial tissue flap adaptation to achieve tension‐free primary closure and restore facial symmetry [9, 10, 17].

A critical surgical consideration in giant parotid resections is the management of postoperative hematoma. The en bloc removal of a 16‐cm lesion results in the creation of a substantial surgical dead space within the parotid bed. In this case, the development of a surgical bed hematoma on Postoperative Day 3 directly coincided with acute, refractory systemic hypertensive spikes. Arterial hypertension, when present in a systemic fashion, has been demonstrated to increase capillary bed perfusion pressure. This, in turn, has been shown to predispose the large, exposed wound surface to microvascular oozing and blood accumulation within the uncollapsed dead space. The prompt surgical re‐exploration, clot evacuation, meticulous electrocautery, placement of closed‐suction drainage, and aggressive pharmacological control of blood pressure resolved the complication without compromising the final functional outcome. This event underscores the imperative for the implementation of multifaceted preventive protocols, encompassing rigorous postoperative blood pressure management, sufficient closed‐suction drainage, and expeditious postoperative clinical monitoring in cases of giant head and neck resections [18].

A comparison of this case with previously published reports of giant parotid PAs (Table 1) reveals both shared features and distinguishing characteristics. While reported cases vary in size, weight, and surgical approaches, the present case is distinguished by sequential, documented disease progression between 2021 and 2025, combined total preservation of facial nerve function (House–Brackmann Grade I), concomitant primary skin remodeling, and successful management of a severe hypertension‐induced postoperative hematoma.

**TABLE 1:** Comparison between giant pleomorphic adenomas of the parotid gland reported in the literature and the present case.

Study	Duration	Size (imaging/clinical)	Specimen size/weight	Surgical approach	Facial nerve outcome	Complications	Histopathology	Follow‐up
Present case (2026)	18 years (2007–2025)	MRI 9.8 × 5.2 × 4.0 cm (2021); CT 14.5 cm/clinical > 15 cm (2025)	16 × 13.5 × 12 cm, 1.76 kg	Superficial parotidectomy with facial nerve preservation + skin remodeling	Preserved, HB grade I	Postoperative hematoma (Day 3), surgical re‐exploration	Benign PA, no atypia	14 months, no recurrence
Tarsitano et al. 2015 [5]	> 30 years	Clinical 35 × 28 cm	33 × 27 × 16 cm, 7.3 kg	Total excision, clear dissection plane, facial nerve avoided	Preserved, HB Grade I	None	Benign PA, negative margins	5 years, no recurrence
Takahama et al. 2008 [19]	> 30 years	Clinical ∼30 cm crossing midline	28 × 20 × 16 cm, 4.0 kg	Total parotidectomy with facial nerve preservation	Preserved, no palsy	None	Benign PA, negative margins	Not specified, no recurrence at last visit
Buenting et al. 1998 [8]	∼20 years	Not specified	26 cm diameter, 6.85 kg	Not specified	Not specified	Not specified	Benign, no malignant transformation	Not specified
Pareek et al. 2022 (*n* = 15) [13]	5–20 years	10 × 6–25 × 22 × 15 cm	2.0–3.5 kg	1 enucleation, 3 superficial, 11 total parotidectomy (+1 neck dissection)	Preserved in 13/15; transient weakness in 2 (recovered < 6 mo)	Not specified	14 benign, 1 malignant (carcinoma ex‐PA)	≥ 6 months, no recurrence
Gobishangar et al. 2023 [10]	22 years	Imaging 23 × 20 × 14 cm	30 × 27 × 18 cm, 2.78 kg	Total/radical parotidectomy + pinna excision	Sacrificed	None reported; preventive tarsorrhaphy	Benign PA	1 year, no recurrence
Jain et al. 2015 [16]	5 years	Clinical 8 × 10 cm	8 × 10 × 12 cm, 1.8 kg	Superficial (superior) lobe excision, modified Blair incision	Preserved	None	Benign PA	3 years, no recurrence
Gupta et al.^∗^ [6]	> 4 years	12 × 8 × 8 cm (CT 11.5 × 7.5 cm)	2.24 kg	Standard submandibular approach^∗^	Not compromised	None	Benign, intact capsule	3 years, no recurrence
Infante‐Cossio et al.^†^ (*n* = 3) [12]	Not specified	Mean 5.7 cm (parapharyngeal)^†^	Not specified	Transparotid‐intraoral, superficial lobe preserved	Preserved in all	None	Not detailed	Not specified, no recurrence

^∗^Submandibular gland case (not parotid); included for size comparison only.

^†^Parapharyngeal‐space series with substantially smaller mean lesion size than the “giant” (> 10 cm) threshold used elsewhere in this comparison; included for surgical‐technique comparison only.

In the largest published review of giant parotid PAs, Schultz‐Coulon analyzed 31 cases and reported a marked female predominance (64.5%), a wide age range (20–40 years), tumor weights ranging from 1 to 27 kg, and malignant transformation in 3 cases (9.7%) [20]. A comparison of this case with previously published reports of giant parotid PAs (Table 1) reveals both shared features and distinguishing characteristics. While reported cases vary in size, weight, and surgical approaches, the present case is distinguished by sequential, documented disease progression between 2021 and 2025, combined total preservation of facial nerve function (House–Brackmann Grade I), concomitant primary skin remodeling, and successful management of a severe hypertension‐induced postoperative hematoma.

This study has several limitations that should be acknowledged. First, given the nature of the study as a single‐case report, the generalizability of our surgical strategy and clinical observations is limited. Second, the patient’s initial refusal of surgery resulted in a 3.5‐year interval during which no clinical or diagnostic surveillance was conducted. This precluded the characterization of intermediate growth dynamics. Third, it is important to note that a repeat preoperative fine‐needle aspiration or core biopsy was not performed in 2025 prior to surgery. Instead, high‐resolution CT imaging and definitive postresection histopathology were used to make a diagnosis. Fourth, the evaluation of facial nerve outcomes was exclusively conducted using the House–Brackmann scale, without the incorporation of validated patient‐reported functional or quality‐of‐life assessment instruments. In conclusion, follow‐up at 3 months and again at approximately 14 months postoperatively confirmed complete functional recovery and no clinical evidence of recurrence; nonetheless, longer‐term surveillance remains imperative, as recurrence of PA may occur many years after excision.

## Author Contributions

Conceptualization: Maria Giulia Cristofaro and Francesco Ferragina; methodology: Francesco Ferragina and Angelo R. Sottile; software: Giuseppe Tarallo and Maria Grazia Ioppolo; validation: Maria Giulia Cristofaro and Francesco Ferragina; formal analysis: Francesco Ferragina; investigation: Angelo R. Sottile, Giuseppe Tarallo, and Maria Grazia Ioppolo; resources: Giuseppe Tarallo; data curation: Francesco Ferragina; writing–original draft preparation: Francesco Ferragina; writing–review and editing: Maria Giulia Cristofaro and Francesco Ferragina; visualization: Maria Giulia Cristofaro; supervision: Maria Giulia Cristofaro and Francesco Ferragina; project administration: Francesco Ferragina; funding acquisition: Maria Giulia Cristofaro.

## Funding

This research received no external funding. Open access publishing facilitated by Universita degli Studi Magna Graecia di Catanzaro, as part of the Wiley ‐ CRUI‐CARE agreement.

## Disclosure

All authors have read and agreed to the published version of the manuscript. All authors agree to be responsible for the content and conclusions of the article.

## Ethics Statement

This study was conducted in accordance with the ethical standards outlined in the Declaration of Helsinki on Medical Protocol and Ethics. The study was approved by the Ethics Committee of Magna Graecia University of Catanzaro (protocol no. 280, dated September 17, 2020), and all patients provided informed consent to participate in the study.

## Consent

Please see the Ethics Statement. Informed consent was obtained from the subjects involved in the study. The patient also provided written informed consent for the publication of clinical images.

## Conflicts of Interest

The authors declare no conflicts of interest.

## Data Availability

The data that support the findings of this study are available on request from the corresponding author. The data are not publicly available due to privacy or ethical restrictions.
